# Sarcophine-Diol Inhibits Expression of COX-2, Inhibits Activity of cPLA_2_, Enhances Degradation of PLA_2_ and PLC_γ_1 and Inhibits Cell Membrane Permeability in Mouse Melanoma B_16_F_10_ Cells 

**DOI:** 10.3390/md10102166

**Published:** 2012-09-28

**Authors:** Pawel T. Szymanski, Pratik Muley, Safwat A. Ahmed, Sherief Khalifa, Hesham Fahmy

**Affiliations:** 1 Department of Pharmaceutical Sciences, College of Pharmacy, South Dakota State University, Brookings, SD 57007, USA; Email: pawel.szymanski@sdstate.edu (P.T.S.); Pratik.Muley@sdstate.edu (P.M.); 2 Department of Pharmacognosy, Faculty of Pharmacy, Suez Canal University, Ismailia 41522, Egypt; Email: safwat_aa@yahoo.com; 3 College of Pharmacy, Qatar University, Doha 02713, Qatar; Email: sherief@qu.edu.qa

**Keywords:** melanoma, sarcophine, sarcophine-diol, skin cancer, melanoma

## Abstract

Sarcophine-diol (SD) is a semi-synthetic derivative of sarcophine with a significant chemopreventive effect against non-melanoma skin cancer both *in vitro* and *in vivo*. Recently, we have studied the effect of SD on melanoma development using the mouse melanoma B_16_F_10_ cell line. In this study, our findings show that SD suppresses cell multiplication and diminishes membrane permeability for ethidium bromide (EB), a model marker used to measure cell permeability for Ca^2+^ ions. SD also decreases protein levels of COX-2, and increases degradation of phospholipases PLA_2_ and PLC_γ_1 and diminishes enzymatic activity of the Ca^2+^-dependent cPLA_2_. This lower membrane permeability for Ca^2+^-ions, associated with SD, is most likely due to the diminished content of lysophosphosphatidylcholine (lysoPC) within cell membranes caused by the effect of SD on PLA_2_. The decrease in diacylglycerol (DAG) and inositol 1,4,5-triphosphate (IP_3_) due to inhibition of PLC_γ_1, leads to the downregulation of Ca^2+^-dependent processes within the cell and also inhibits the formation of tumors. These findings support our previous data suggesting that SD may have significant potential in the treatment of melanoma.

## 1. Introduction

**Figure 1:**
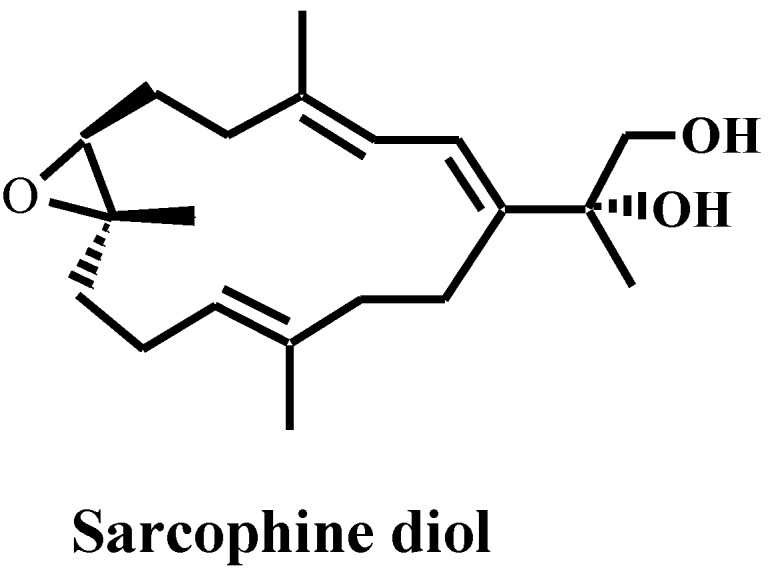
Structure of sarcophine-diol (SD).

Recently, there has been a considerable interest in the use of natural marine products for inhibiting skin cancer development [1,2,3,4,5,6,7]. In the last decade, our laboratory has taken the lead to study chemopreventive skin cancer as well as the anti-cancer effects of sarcophine diol (SD). SD (Figure 1) is a semi-synthetic derivative of sarcophine which is a cembranolide isolated from the Red Sea coral *Sarcophyton glaucum* [3].

Our studies demonstrated that SD is a very promising chemopreventive agent against chemically-induced and UV-induced non-melanoma skin cancer in both animal and cell-culture models. Topical application of SD was found to enhance expression levels of cleaved-Caspase-3 and -8, molecular markers for apoptosis in chemically-induced skin cancer in female CD-1 mice [1]. We also demonstrated that SD treatment inhibits skin tumor development in UV-induced skin cancer in SKH-1 hairless mice [4]. The latter investigations also revealed that topical application of SD enhances cellular levels of cleaved-Caspase-3 and -8, and increases the rates of DNA fragmentation in skin cells isolated from these mice [4]. Our subsequent studies demonstrated that SD, in a concentration-dependent manner, decreases cell viability in human epidermoid carcinoma A431 cell line [8]. These studies also showed that SD treatment inhibits the incorporation of the thymidine analogue 5-bromo-2′-deoxyuridine (BrdU) in *de novo* synthetized DNA. Moreover, SD treatment was found to enhance fragmentation of DNA in the same human epidermoid carcinoma A431 cell line and increases expression level of caspase-3 through activation of upstream caspase-8 in these cancer cells [8].

Recently, we extended our research to study the effect of SD on melanoma development using the mouse melanoma B_16_F_10_ cell line since numbers of new melanoma diagnoses has been on the rise [9]. Our results showed that SD inhibits the *de novo* DNA synthesis and enhances DNA fragmentation. SD also inhibits the expression of several biomarkers of proliferation and apoptosis [10]. It inhibits the levels of signal transducer and activator of transcription protein STAT-3 and cyclin D1, an activator of cyclin-dependent kinase 4 (cdk4), enhances the levels of tumor suppressor protein p53, and stimulates cleavage of the nuclear poly-(ADP-ribose) polymerase PARP. It also enhances both protein levels as well as the enzymatic activities of caspase-3, -8 and -9. These findings, in addition to inhibition of cell viability, suggest that SD inhibits melanoma cell proliferation by arresting the cell-division cycle in the Go quiescent phase and also activates two pathways involved in programmed cell death (*i.e.*, extrinsic and intrinsic). 

In this study, results showed that treatment with SD suppresses cell multiplication rather than death from *de novo* generated cells. SD was found to diminish membrane permeability for ethidium bromide (EB), which is a model marker for cell permeability for Ca^2+^ ions. SD was also found to decrease protein levels of cyclooxygenase-2 (Cox-2), increase degradation of phospholipase A_2_ (PLA_2_), phospholipase Cgamma1 (PLC_γ_1), and diminish enzymatic activity of Ca^2+^-dependent phospholipase A_2_ (cPLA_2_). This lower membrane permeability for Ca^2+^ ions in SD treated cells, is proposed to be due to the diminished content of lysophosphosphatidylcholine (lysoPC) within cell membranes due to inhibiton of PLA_2_ by SD, which is related to its effect on the caspases [11,12]. It also suggests that the lower contents of diacylglycerol (DAG) and inositol 1,4,5-triphosphate (IP_3_) caused by inhibition of PLC_γ_1 by SD leads to inhibition of the Ca^2+^-dependent processes within the cell.

## 2. Results and Discussion

### 2.1. Wound Healing Assay

**Figure 2:**
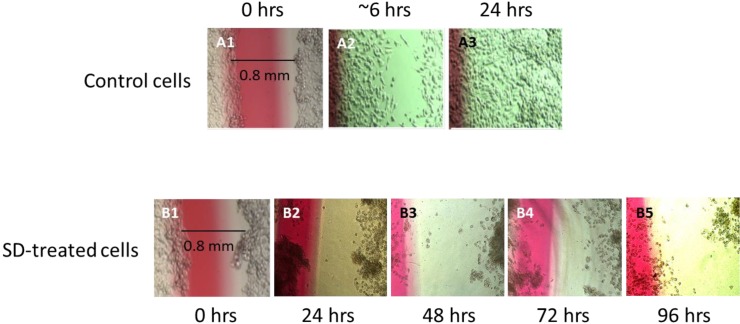
Light microscopy images of wound healing in untreated control melanoma cells during 6 h and 24 h of incubation, and in cells treated with a 250 µM concentration of SD for 24 h, 48 h, 72 h and 96 h, respectively.

In a view of our previous investigation showing that SD stimulates signaling pathways that lead to apoptosis in mouse melanoma B_16_F_10_ cells [10], we were interested to further extend these studies. A wound healing assay was performed. As illustrated in Figure 2, a wound of <1 mm in width in untreated control cells is partially covered as early as the first 6 h of incubation, and complete healing was observed after 24 h. A similar wound in cells treated with 250 µM concentration of SD was not repaired during the first 24 h or even after 96 h suggesting that cells treated with SD do not duplicate. 

### 2.2. SD Inhibits Cell Multiplication

As illustrated in Table 1, the total living cell numbers in untreated control cell samples increased by ~13.7-fold during 72 h incubation period. The total protein content in untreated control cells increased by ~11.6-fold during 72 h incubation. Treatment of the same cells with increasing concentrations of SD inhibits cell duplication in a dose-dependent manner. This is reflected in the total number of living cells and the total cellular protein content. Maximal inhibition of cell growth in terms of both total living cell numbers and total cellular protein content compared to untreated controls was observed in cells treated with 250 µM concentration of SD for 72 h of treatment.

**Table 1:** The effects of the increasing concentrations of SD (from 0 µM to 250 µM) during 72 h incubation on the total cellular protein contents and total living cells number. Values are means ± Standard Deviation (St. Dev.), and (*) values with statistical difference (*p*<0.05) as compared to untreated control cells after 72 h (0 µM).

	Time 0	0 µM	62.5 µM	125 µM	250 µM
Total protein content (µg)	26.62 ± 5.49, *n* = 5	309.41 ± 43.93, *n* = 5	201.67 ± 35.35, *n* = 5 *	165.83 ± 19.57, *n* = 5 *	38.57 ± 7.32, *n* = 5 *
Total living cells	1.86 × 10^5^ ± 4.7 × 10^4^, *n* = 5	2.4 × 10^6^ ± 4.5 ×10^5^, *n* = 5	1.68 × 10^6^ ± 2.7 × 10^5^, *n* = 5 *	1.11 × 10^6^ ± 1.9 × 10^5^, *n* = 5 *	2.3 × 10^5^ ± 5.5 × 10^4^, *n* = 5 *

**Figure 3:**
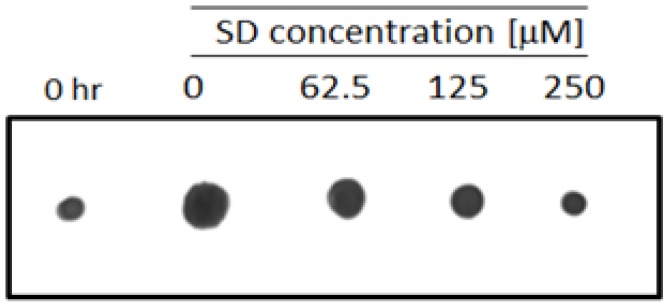
Image of the Amido-Black Dot-blot of total protein extracts loaded from the same concentration of melanoma cell samples growing in the presence of increasing concentrations of SD for 72h.

To illustrate the inhibitory effect of SD treatment on melanoma cell multiplication, cell extracts were loaded on nitrocellulose membranes and stained with Amido-black. Figure 3 shows images from the attached cell samples loaded from the same concentration. Protein content in cells treated with a 250 µM concentration of SD for 72 h is nearly the same as untreated control cells at time “0”, suggesting that SD most likely inhibits wound healing due to inhibition of cell multiplication, rather than directly inhibiting the invasiveness of the cells. 

### 2.3. SD Inhibits Cell Membrane Permeability

**Figure 4:**
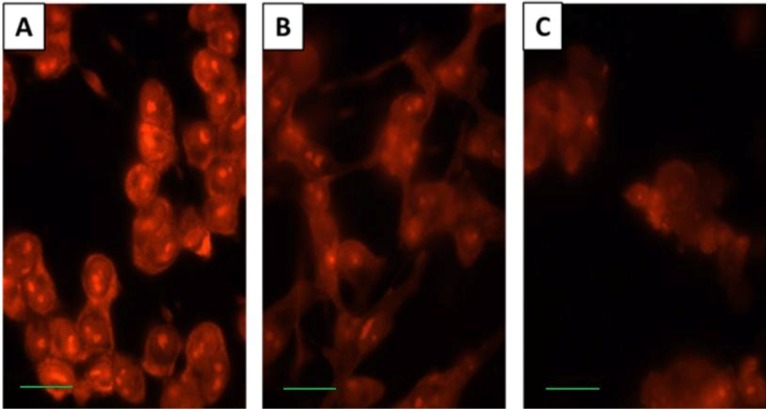
Fluorescence microscopy images of melanoma cells stained with EB. Intact untreated control cells (A) and cells treated with 250 µM concentration of SD for 24 h and 72 h, respectively (B and C). Scale bars are 10 µm in length. These images are representative of 5 replicates.

The effect of SD on membrane permeability was studied. It is commonly accepted that free levels of Ca^2+^ ions within the cytoplasm play a crucial role in the regulation of several vital processes [13,14]. Nucleic acids in untreated control cells and cells treated with a 250 µM concentration of SD for 24 h and 72 h were stained with EB. EB is a low molecular weight, membrane impermeable fluorescent dye that binds exclusively to nucleic acids [15]. As illustrated in Figure 4A, untreated control cells display strong fluorescence intensity inside cells, suggesting high membrane permeability for EB. Treatment with SD for 24 h decreases nucleic acid staining with EB (Figure 4B). Only marginal staining of the cells with EB was detected in cells treated with SD for 72 h (Figure 4C). This suggests that SD treatment reduces permeability of the cell membrane for Ca^2+^ ions. This supports the hypothesis that inhibition of cell duplication by SD may result from reduced influx of Ca^2+^ ions from the extracellular space to the cytoplasm. 

### 2.4. SD Inhibits Expression Levels of Cox-2 and Enhances Degradation of PLA2 and PLC_γ_1

**Figure 5:**
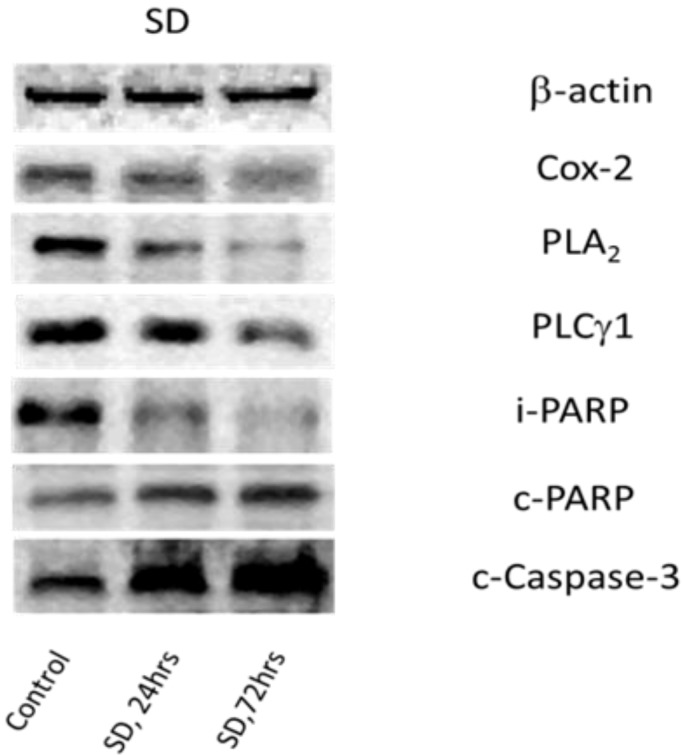
Western blots of some molecular biomarkers for cell proliferation and apoptosis in melanoma cells treated with 250 µM SD for 24 h and 72 h.

Cox-2 is an enzyme that converts arachidonic acid released from the membrane-associated phospholipids, particularly phosphatidylcholine (PC) by Ca^2+^-dependent phospholipase A_2_ (cPLA_2_) [16,17] to prostaglandins [18,19]. While abundant at sites of inflammation [20], Cox-2 was found to be upregulated in various carcinomas [1,21,22]. As inflammation accompanies cell proliferation [23,24], and SD shows anti-tumor activity in many types of skin cancers [1,3,4,5,7,10] it was of interest to determine the SD effects on the protein level of Cox-2. Melanoma cells were incubated in the presence and absence of SD, and after harvesting cells, expression levels of Cox-2 in cellular protein extracts were determined. As illustrated in Figure 5 and Table 2, exposure of cells to a 250 µM concentration of SD produces a time-dependent decline in the expression levels of Cox-2. Maximal inhibition (roughly 70%) was observed during 72 h. A smaller inhibition (roughly 40%) was observed after 24 h.

There is growing data showing that cancer cells display high contents of both cPLA_2_ [11] and PLC_γ_1 [12,25], and that their content declines during apoptosis due to elevation of proteolytic activities of caspases, mostly of type II [11,12,25]. The effects of SD on degradation of PLA_2_ and PLC_γ_1 were studied. Melanoma cells were incubated in the presence or absence of SD, and after harvesting the cells, the relative content of non-degraded isoforms of these two phospholipases in cellular protein extracts was determined. As illustrated in Figure 5 and Table 2, exposure of the cells to a 250 µM concentration of SD produces a time-dependent decline in the expression levels of intact PLA_2_ and PLC_γ_1. The highest reduction in levels of intact PLA_2_ and PLC_γ_1 (roughly 70%) was observed during 72 h exposure while a smaller degradation of PLA_2_ and PLC_γ_1 (roughly 40%) was observed after 24 h.

To confirm that lower levels of PLA_2_ and PLC_γ_1 result from specific protein degradation involving cleaved-Caspase-3, the protein level of cleaved-Caspase-3 and its major substrate, involved in programmed apoptotic cell death cascades such as PARP, was measured. As illustrated in Figure 5 and Table 2, treatment with a 250 µM concentration of SD at 24 h and 72 h increased, in a time-dependent manner, the expression levels of cleaved-Caspase-3. A 40% and 90% increase was observed after 24 and 72 h, respectively. Using the same Western blot analysis, it was observed that treatment of cells with 250 µM concentration of SD for 24 h and 72 h also produced a significant time-dependent decline in the level of intact-PARP coupled with a significant time-dependent increase in the level of cleaved-PARP. The levels of intact-PARP decreased by 30% and 50% during 24 h and 72 h treatment with SD, respectively, while levels of cleaved-PARP increased by 40% and 100% during the same time period.

**Table 2:** The effect of 250 µM SD on the relative content of protein biomarkers for cell proliferation and apoptosis. Values are means ± Standard Deviation (St. Dev.), and (*) values with statistical difference (*p*<0.05) compared to untreated control cells.

	Control	24 h, SD	72 h, SD
Cox-2	100	56.81 ± 10.22, *n* = 4, *	27.62 ± 6.85, *n* = 4, *
PLA_2_	100	57.41 ± 14.63, *n* = 7, *	30.58 ± 9.67, *n* = 7, *
PLC_γ_1	100	59.20 ± 6.53, *n* = 8, *	34.44 ± 10.05, *n* = 8, *
i-PARP	100	68.51 ± 12.06, *n* = 4, *	50.16 ± 13.47, *n* = 4, *
c-PARP	100	150.41 ± 26.31, *n* = 4, *	226.32 ± 35.27, *n* = 4, *
c-Casp-3	100	143.1 ± 20.26, *n* = 6, *	197.22 ± 21.75, *n* = 6, *

### 2.5. SD Inhibits Enzymatic Activity of cPLA_2_

**Figure 6:**
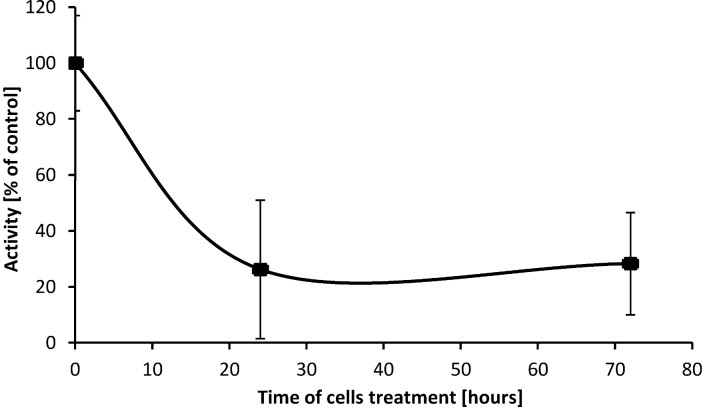
Enzymatic activities of cPLA_2_ in protein extracts from melanoma cells treated with a 250 µM concentration of SD for 24h and 72h.

As cPLA_2_ catalyzes hydrolysis and release of arachidonic acids from position C-2 in esterified PC [26], leading to elevation of lysophosphatidylcholine (lysoPC) in the cell membrane [27], the effects of SD treatment on enzymatic activity of this phospholipase was investigated. Using the synthetic substrate arachidonyl thio-PC and specific inhibitors for iPLA_2_ and sPLA_2_, the enzymatic activity of cPLA_2_ in protein extracts from untreated control cells and cells treated with SD for 24 h and 72 h was determined. As illustrated in Figure 6, exposure of the cells to a 250 µM concentration of SD produces a time-dependent decline in enzymatic activity of cPLA_2_. The lowest enzymatic activity (70%), measured as rate of hydrolysis of arachidonyl thio-PC by cPLA_2_, was observed at a 250 µM concentration of SD and 24 h of incubation. Further incubation time does not decrease the activity any further.

### 2.6. Discussion

In our recent study [10], SD was found to inhibit DNA synthesis, determined by incorporation of BrdU on *de novo* synthetized DNA, and simultaneously stimulate DNA fragmentation in B_16_F_10 _melanoma cells. SD was also found to enhance degradation of PARP that appears to be involved in DNA repair and helps cells to maintain their viability [28]. Inactivation of PARP can be facilitated by many ICE-like caspases, and PARP is one of the main cleavage targets for Caspase-3 *in situ* [29,30,31,32,33,34]. The same study also demonstrated that SD treatment increases the protein level of cleaved-Caspase-3 and its enzymatic activity. As a result, cells treated with SD are characterized as having lower content of intact-PARP and higher content of the cleaved-PARP isoform, as compared to untreated control cells. Data also shows that SD inhibits expression of STAT-3, a protein that plays a role in the expression of genes that are involved in cell survival, proliferation, chemo-resistance and angiogenesis [35,36]. Moreover, SD treatment was also demonstrated to increase expression of the tumor suppressor protein p53, which is known to activate many pro-apoptotic factors, including BAX, Bak, Fas/APO, PiDD, *etc.* [37,38].

To extend our studies to further explore the inhibitory effect of SD on melanoma and to identify a possible mechanism of action at the molecular level, our investigation showed that SD inhibits cell duplication. This notion is based on findings that cells treated with SD display the same fraction of floating cells/dead cells as untreated control cells. 

Treatment with SD inhibits staining of our cells with EB suggesting that it inhibits permeability to Ca^2+^ and thus inhibits Ca^2+^-dependent processes within cells. It is commonly accepted that cyclooxygenase-2 (Cox-2) is undetectable in most normal tissues. It is an inducible enzyme, becoming abundant at sites of inflammation [20]. Recently, it has been shown to be upregulated in various carcinomas [21,22]. In this investigation, SD was found to decrease cellular levels of Cox-2. Cox-2 converts arachidonic acid that is released by cPLA_2_ from phospholipids, particularly phosphatidylcholine (PC) [16,17] to prostaglandin H2 (PGH2), a precursor of the series-2 prostanoids [18,19]. SD treatment stimulates inactivation of cPLA_2_. High activity of cPLA_2_ in tumor cells [11] increases levels of lysoPC within the cell membrane. Several studies have demonstrated that a higher content of lysoPC makes plasmalemma more fluid [27] and permeable to intracellular mediators such as Ca^2+^ ions [39,40]. Membrane permeability is implicated in DNA damage and repair [41,42] and there is growing evidence showing that high content of lysoPC accompanies inflammation [21,22], cell proliferation [43,44] and cancer development [11,45]. 

Untreated control tumor cells display high activities of cPLA_2_ and PLC_γ_1. High activity of cPLA_2_ increases cell membrane fluidity due to higher content of lysoPC in membranes and causing free Ca^2+^ ions concentration in the cytoplasm to rise. Higher content of arachidonic acid induces activity of Cox-2 and the production of prostaglandins. Higher activity of PLC_γ_1 produces DAG, an activator of protein kinase C (PKC) and inositol-1,4,5-triphosphate (IP_3_). The IP_3_ activates Ca^2+^ ion channels in the endoplasmic reticulum that enhances the level of free Ca^2+^ ions in the cytoplasm [46,47,48]. SD was found to stimulate degradation and cleavage of PLC_γ_1 and cPLA2. This suggests that SD is involved in the regulation of two important signaling mediators derived from membrane-associated phosphatidylinositol 4,5-biphosphate (PIP_2_): diacylglycerol (DAG) and inositol 1,4,5-triphosphate (IP_3_) that play a role in several biochemical processes. DAG acts similarly to phorbol esters, which are well known tumor promoters that activate protein kinase C (PKC) cascades [31,32] and was found to be a strong enhancer of cell proliferation [46]. PKC signaling pathways are involved in phosphorylation of many proteins *in vitro* and are upregulated in different types of cancers including cutaneous melanoma [46]. Thus the fact that SD stimulates cleavage and degradation of PLC_γ_1 suggests that SD also inhibit the synthesis of an endogenous tumor-promoting factor (DAG). 

## 3. Experimental Section

### 3.1. Chemical and Reagents

The gel apparatus and material for electrophoresis were purchased from Hoefer (San Francisco, CA) and Bio-Rad (Richmond, CA). All commonly used reagents, 10,000 units of Penicillin, 10 mg Streptomycin stock solutions and Trypsin-EDTA 10× were purchased from Sigma-Aldrich (St. Louis, MO). Alexa Fluor488-phalloidin was purchased from Invitrogen (Eugene, CA). 0.4% Trypan-blue solution was purchased from MidSci Amersco (Solon, OH) and 4% paraformaldehyde in PBS buffer solution was purchased from USB Corporation (Cleveland, OH). The Elisa-type kit for determination of enzymatic activity of cPLA_2_ was purchased from Cayman Chemical Company (Ann Arbor, MI). Mouse melanoma B_16_F_10_ cell line and Dulbecco’s Modified Eagle’s Media (DMEM) supplemented with L-glutamine and Fetal Bovine Serum (FBS) were purchased from the American Type Culture Collection ATCC (Manassas, VA). Rabbit polyclonal antibodies against Cox-2 and cleaved-PARP were purchased from Cell Signaling (Danvers, MA). Rabbit anti-PLA_2_ antibody and mouse monoclonal anti-PLC_γ_1 antibodies were purchased from Abcam, Inc. (Cambridge, MA). Mouse monoclonal anti-β-actin antibody was purchased from Sigma (St. Louis, MO), and the anti-intact PARP mouse monoclonal antibody was purchased from Santa Cruz Biotechnology, Inc., (Santa Cruz, CA). Goat anti-mouse and goat anti-rabbit HRP-conjugated secondary antibodies and nitrocellulose transfer membranes with a pore size of 0.22 µm in diameter were purchased from Santa Cruz Biotechnology, Inc., (Santa Cruz, CA). 

### 3.2. Semi-Synthesis of Sarcophine-Diol (SD)

Sarcophine was isolated from the Red Sea soft coral *Sarcophyton glaucum* as described previously [3,4], and converted to sarcophine-diol (SD) using a two-step reaction according to our published procedures [3]. SD was dissolved in dimethyl sulfoxide (DMSO) to prepare a 50 mM stock solution. In all culture media, the final concentration of DMSO was 0.5% including untreated controls, whereas the final concentration of SD ranges from 0 µM to 250 µM.

### 3.3. Cell Culture

Under standard conditions, cells were grown in DMEM media supplemented with 10% FBS and 1% solution containing 100 U of penicillin/mL of media and 100 mg streptomycin/mL of media. Cells were incubated in 37 °C in a humidified atmosphere containing 95% O_2_ and 5% CO_2_. 

### 3.4. Wound Healing Assay

A wound healing assay was used to compare the relative proliferation of control cells and cells treated with SD. ~7 × 10^4^ cells were seeded in growth media in a polystyrene petri dish (3.5 cm diameter) and incubated for 24–36 h until cells completely covered the surface of the dish. A wound of <1 mm in width was made across the dish using a rubber scraper, and the scraped/detached cells were gently removed from the dish. Fresh media was poured into each dish, and cells were incubated in the presence of a 250 µM concentration of SD for 24–96 h. A control experiment without SD was also carried out. After each 24 h cycle, wound healing was examined using light microscopy.

### 3.5. Cell Membrane Permeability

Ethidium bromide (EB) was used to assess the membrane permeability for Ca^2+^-ions. ~2–4 × 10^3^ cells suspended in 0.75 mL of DMEM growth media were seeded in 24 well plates with removable glass cover-slips. Cells were incubated for 24 h. Once cells became attached to the surface, the old media was exchanged with fresh media containing 0.5% DMSO (control cells) or SD (250 µM), and cells were incubated for 24 h and 72 h. At the end of incubation period, cells were quickly washed with PBS (3 × 0.5 mL each time), and then stained with EB in 0.5 mL PBS (10 µg EB per mL, final concentration) at room temperature for 15 min with constant gentle rocking. Cells were then thoroughly washed with PBS to remove the excess of dye (4 times with 1 mL PBS, 5 min each). Cells were removed from the well and the cover-slips were mounted on a specimen glass slide. The EB stained nucleic acids were examined under a model A × 10 Zeiss fluorescence microscope (Jena, Germany) connected to AxioVison Rel.4.7 software via a Zeiss camera model AxioCam MRC5. 

### 3.6. Determination of Cell Multiplication and Cellular Protein Content

A fixed number of cells were seeded in each well of a 6 well plate (~0.8 × 10^5^ cells/5 mL well), and incubated for 24 h. Upon removal of the old media, cells in fresh media were incubated in the presence and absence of different concentrations of SD ranging from 0 µM to 250 µM for 72 h. Then, both the growth media and the attached cells were collected, and these two cell-containing fractions were used for determining the total cell number and also for total cellular protein contents. 

To determine total protein contents and to perform Western blot analysis of Cox-2 and PARPs, cells were detached from the surface of a polysterene flask by short exposure to a minimal concentration of trypsin. Immediately after detachment, the cell suspension was diluted with 10-fold excess of growth media to inhibit further proteolysis by trypsin. To determine the status of degradation of PLA_2_ and PLC_γ_1 as well as enzymatic activity of cPLA_2_ in cellular extracts, cells were detached from the flask using a rubber scraper. The latter procedure minimizes cleavage of these phospholipases by cellular proteases, including Caspases [11,12]. Immediately after detachment, the cells were pelleted in 4 °C at 1700 rpm for 5 min. Then, the cell pellets were thoroughly washed with PBS to remove contamination from FBS, and this procedure was repeated 3 times. The final cell pellets were solubilized in 3–5 volume of the appropriate ice-cold extraction buffer by passing through a pipette tip several times. Cell lysates were then kept in an ice-cold extraction buffer for 10–15 min, and spun down at 4 °C at 14,000 rpm for 20 min. Supernatants were collected and stored at −70 °C for further determinations. 

For determination of the expression levels of Cox-2, PARPs and cleaved-Caspase-3, the extraction buffer composition was 20 mM Tris(hydroxymethyl)aminomethane (Tris)-HCl pH 7.8, 150 mM NaCl, 1% octylphenoxypolyethoxyethanol (Triton X-100), 1% sodium deoxycholate, 0.1% sodium dodecyl sulfate (SDS), 2 mM ethylenediaminetetraacetic acid (EDTA), 2 mM ethylenebis(oxyethylenenitrilo)tetraacetic acid (EGTA), 2 mM sodium azide (NaN_3_), 2 mM dithiothreitol (DTT). For determination of phospholipases, the extraction buffer composition was: 1× PBS buffer, pH 7.4 supplemented 1 mM EDTA. To minimize protein degradation during extraction, these buffers were supplemented with a protease inhibitor cocktail according to the manufacturer protocol (Roche Diagnostic GmbH, Mannheim, Germany). Specifically, one tablet was diluted in 1 ml of double distilled water and added to 7 mL of extraction buffer. 

### 3.7. Western Blot Analysis

A total cell protein extracts (15–20 µg protein/well) were resolved in 8.5% SDS-PAGE (having a total monomer to cross-linker ratio of 29:1). Once separated, proteins were transferred in an electric field into a nitrocellulose membrane and identified by the protein anti-bodies using the following procedure: The nonspecific binding sites were blocked by Tris-buffered saline pH 7.5 supplemented with 0.05% polyoxyethylenesorbitol monolaurate (Tween 20) and 5% non-fat milk at room temperature for 1 h. Then, membranes were probed with primary antibodies at 4 °C overnight using a dilution of 1:1000 for β-actin, Cox-2 and PARPs, and 1:500 for PLA_2_ and PLC_γ_1. Primary antibodies were diluted in Tris-buffer saline pH 7.5 containing 0.05% Tween and 1% non-fat milk. Membranes were thoroughly washed with Tris-buffer saline pH 7.5 containing 0.05% Tween 20, and then secondary antibodies were applied. All secondary antibodies were diluted in Tris-buffer saline supplemented with 0.05% Tween 20, using a dilutions of 1:2000 for detection of β-actin, Cox-2 and PARPs, and 1:1000 for PLA_2_ and PLC_γ_1. Membranes were exposed to secondary antibodies at room temperature for 1.5 h. After several washes with Tris-buffer saline pH 7.5 the protein bands were visualized by the ELC detection kit (Santa Cruz Biotechnology, Inc., Santa Cruz, CA) and quantified using UVP Biochem Gel Documentation System (UVP, Inc., Upland, CA). Equal protein loading was ensured by re-probing each membrane with anti-β-actin antibody. 

### 3.8. Determination of Enzymatic Activity of cPLA_2_

An Elisa-type kit (Cayman Chemical Company, Ann Arbor, MI) was used to determine enzymatic activity of the Ca^2+^-dependent phospholipase A_2_ (cPLA_2_) in the protein extracts from cells that grow in the presence and absence of SD. Determination of cPLA_2_ activity was based on detection of free thio-arachidonic acid released from the synthetic arachidonyl thio-PC by cPLA_2_ using Ellman’s reagent. To inhibit the Ca^2+^-independent PLA_2_ (iPLA_2_) and the secretory PLA_2_ (sPLA_2_) activities, all reaction mixtures were supplemented in a 5 µM concentration of bromophenol lactone (BEL) and a 3 µM of thioetheramide-PC, respectively. Cellular protein extracts in concentrations of 3.5 mg/mL in 0.2 mL of reaction buffer containing arachidonyl thio-PC (50 µM final concentration) were incubated at room temperature for 1 h. The reaction was stopped by adding 5,5′-dithio-*bis*-(2-nitrobenzoic acid) (DNTB) in the EGTA/Tris-HCl buffer solution to the incubation mixture. Absorbance of the samples was read at 405 nm. The manufacturer assay buffer consisted of: 80 mM 4-(2-hydroxyethyl)piperazine-1-ethansulfonic acid (HEPES) pH 7.4, 150 mM NaCl, 10 mM CaCl_2_, 4 mM Triton X-100, 30% glycerol, and 1 mg/mL BSA.

### 3.9. Staining of the Cells with Trypan-Blue

A quick Trypan-blue exclusion staining method was also used to determine cell viability. Cell suspension in growth media was diluted 2-fold with a 0.4% Trypan-blue solution. The number of living cells were determined using a Cellometer Auto T4 Plus Cell Counter (Nexcelom Bioscience LLC, Lawrence, MA) equipped with Cellometer Automated Cell Counts software. 

### 3.10. Determination of Protein Content

Total protein content in the cell extracts was determined colorimetrically using a detergent compatible bicinchoninic acid (BCA) protein assay kit according to the manufacturer protocol (Pierce, BCA, Rockford, IL) using bovine serum albumin (BSA) as a standard.

### 3.11. Statistical Analysis

All values are expressed as means ± Standard Deviation (St. Dev.) Student’s *t-test* is used for statistical analysis of Table 2 and ANOVA test was used for statistical analysis for Table 1 and a confidence level of *p* < 0.05 was chosen as an indication of statistical difference.

## 4. Conclusions

SD suppresses multiplication of melanoma B_16_F_10_ cells, diminishes permeability for Ca^2+^ ions and decreases protein levels of COX-2, and increases degradation of phospholipases PLA_2_ and PLC_γ_1 and diminishes enzymatic activity of the Ca^2+^-dependent cPLA_2_. These finding supports our previous data suggesting that sarcophine-diol may have a significant potential in treatment of melanoma.
